# Diabetes Distress Among the Roma Population From a Tertiary Care Center in Romania

**DOI:** 10.7759/cureus.60348

**Published:** 2024-05-15

**Authors:** Andrada Cosoreanu, Emilia Rusu, Doina Andrada Mihai, Florin Rusu, Ileana Pantea, Ioana Paunica, Ioana Ungureanu, Gabriela Radulian

**Affiliations:** 1 Diabetes, University of Medicine and Pharmacy "Carol Davila" Bucharest, Bucharest, ROU; 2 Urology, “Dr. Carol Davila” Central Military Emergency University Hospital, Bucharest, ROU; 3 Diabetes, Transilvania University of Brasov, Brasov, ROU; 4 Diabetes, "Nicolae Malaxa" Clinical Hospital, Bucharest, ROU

**Keywords:** socioeconomic status, health-related quality of life, emotional impact, roma population, diabetes-related distress

## Abstract

Background

Distress in patients with diabetes is a condition that has received significant attention in recent years; however, data regarding the psychological assessment and the impact of the emotional burden of diabetes among the Roma population are still scarce in the medical literature.

Material and methods

We conducted an observational, transversal study that included 310 adult patients with diabetes mellitus, aged between 18 and 85 years old, of which the majority (61%) were women; patients were selected from a tertiary hospital providing diabetes care; diabetes distress was evaluated using a standardized questionnaire, the diabetes distress scale (DDS), validated on Romanian patients.

Results

In the study population, a great proportion of patients showed diabetes distress, with 24.8% (N=82) having moderate distress and 29.7% (N=121) having severe distress. In the Caucasian group, there were significantly more patients without distress than in the Roma patients,while on the contrary, more Roma patients experienced severe distress compared to the opposite group (64.5%, N=78 versus 35.5%, N=43). In the Caucasian group, a statistical significance was observed regarding interpersonal distress, with Caucasian women having a higher score than men. Concerning the Roma patients, total DDS and all subscales´ scores were statistically significant, with Roma women having higher scores than men. A statistical significance was observed between ethnicity and diabetes distress scores, with the Roma population having higher median values than Caucasian patients. It was also demonstrated that the lack of education, a higher diabetes evolution, and a higher glycated hemoglobin (HbA1c) level (above 8%) have influenced the risk of severe DDS in the Caucasian group, while in the Roma patients, employment status (being unemployed) represents a risk factor for severe DDS.

Conclusion

The Roma patients included in our study experienced higher distress scores compared to Caucasians. These results are substantial as they emphasize the need to include the evaluation of diabetes distress in clinical practice to facilitate the early initiation of intervention measures. There is nevertheless limited data regarding this particular ethnic group; therefore, further research is still needed.

## Introduction

Distress in patients with diabetes is a condition that has received significant attention in recent years, as it has been observed that they encounter various inconveniences related to the multitude of self-management responsibilities, such as lifestyle optimization, pharmacologic therapy, self-monitoring of blood glucose, or self-management of hypoglycemia. It can be defined as a state of stress, frustration, and overwhelmingness related to this routine. Apart from this, concurrent mental health conditions, such as anxiety or depression may also complicate self-care [1,2].

Findings in the medical literature suggested that approximately one-third of adults with diabetes suffer from psychological distress and that elevated distress leads to higher glycated hemoglobin (HbA1c) levels, poorer glycemic control, and impaired behavioral self-management [3,4].

As part of increasing health-related quality of life (HRQoL), psychosocial care is extremely important and along with depression, anxiety, or disordered eating behavior, screening for diabetes distress has become an essential parameter to evaluate at least annually, according to the American Diabetes Association [5]. For this reason, the importance of screening not only at the time of diagnosis but also periodically thereafter has been emphasized, as a potential integral part of diabetes management, considering validated surveys such as the Problem Areas in Diabetes Scale (PAID), Questionnaire on Stress in Patients with Diabetes-Revised (QSD-R) or the diabetes distress scale (DDS) [2,3,5].

The DDS, developed by Polonsky et al., represents a valuable screening tool for diabetes distress that has been used worldwide, offering significant reliability and consistent validity and being applicable to patients from different ethnic groups [6]. Therefore, although originally developed in English, it has been translated into many different languages such as Polish, Arabic, Portuguese, Danish, Chinese, Persian, Norwegian, Malaysian, Indonesian, Thai, Indian, and Bengali [7].

The DDS has also been translated and validated to be applied to adult Romanian patients with diabetes (DDS-RO), due to its excellent psychometric properties, suggesting strong reliability and proving its utility as a screening tool for assessing the emotional burden [8]. However, the distress levels among Romanian diabetes patients appeared to be lower in comparison to the thresholds set by Polonsky [6], indicating that diabetes may not be perceived as a significant source of distress in their daily lives [8].

In line with existing research findings, the DDS-RO exhibited a positive association with poorer diabetes management, as indicated by elevated HbA1c levels. Moreover, the DDS-RO showed promising predictive capabilities concerning depressive symptoms, specifically, the emotional strain of diabetes and distress related to interpersonal relationships, notably with family and friends, which displayed the highest correlations with depression. This implies that diabetes negatively affects an individual's perceived social interactions, potentially leading to depressive tendencies. In terms of health-related behaviors, DDS-RO demonstrated its strongest correlations with dietary habits, physical activity levels, and self-monitoring practices [8].

In Romania, one of the most numerous ethnic minorities is represented by the Roma population, the 2021 National Census describes as many as 569 000 members [9]; thus, a topical issue that has raised interest in the medical literature during the last years is the quality of life among this particular ethnic group. Historically, the Roma population has faced inequality in terms of access to health services based on racial origins and socioeconomic status which leads to certain characteristics of their health, including a higher prevalence of communicable and non-communicable diseases as well [10,11]. However, data regarding the psychological assessment and the impact of the emotional burden of diabetes among them are still scarce in the medical literature.

Therefore, our paper aimed to determine the distress level using the DDS-RO in a population with diabetes from Romania, evaluate the correlation with socioeconomic and demographic factors, and assess the differences between the Roma patients and the control group of Caucasians.

## Materials and methods

Trial design

We conducted an observational, transversal study during October 2022 and December 2023.

Participants

We evaluated 310 adult patients with diabetes mellitus, aged between 18 and 85 years old, attending a tertiary hospital providing diabetes care, “Nicolae Malaxa” Clinical Hospital in Bucharest, Romania.

Patients recruitment

Inclusion Criteria

The study consisted of patients diagnosed with type 1 diabetes mellitus (T1DM) or type 2 diabetes mellitus (T2DM), with communication skills and the ability to understand to fill in the questionnaire. Before completing the questionnaire, all participants agreed to participate and signed an informed consent.

Exclusion Criteria

The inability to complete the form, impaired communication and understanding capacity, and rejection of signing the informed consent represent exclusion criteria.

The study was approved by the Ethical Committee for Clinical Studies of the "Nicolae Malaxa" Clinical Hospital, with registration number 75/2022.

Estimation of the sample size

Estimation of the sample size for this study was determined with the assumption that data is normally distributed and the population (N) was very large. The sample size (n) was calculated by using the following statistical formula, n=$Z_{1-ᾁ}^{2}$(p (1-p)/D^2^), including the data from the 2021 Romanian National Census regarding the number of the Roma population in Bucharest and the prevalence of diabetes distress from previously published studies [9]. Therefore, the estimated minimum sample size for our study was 138 Roma patients.

Data collection

Data collection consisted of a questionnaire with three distinct sections including the Romanian version of the DDS, socioeconomic and demographic data, as well as laboratory findings.

Prior to completing the questionnaire, patients received information about the study's objectives and their right to withdraw at any stage, and if needed, assistance from a trained individual was offered for questionnaire completion. Responders were hospitalized patients, either with continuous hospitalization (a minimum of three days of hospitalization) or with one-day hospitalization. The patients received the survey which contained the questions of the DDS-RO and the information regarding their socioeconomic and demographic status. The laboratory parameters were collected from their medical charts.

Evaluation instruments

The DDS involves 17 items to evaluate the psychological concerns of this disease, consisting not only of a total score, but also four additional subscales to assess the emotional burden, physician-related distress, regimen-related distress, and interpersonal distress. Each item can be scored using a six-point scale, ranging from 1 (no problem) to 6 (serious problem). To calculate the scores, the numbers indicated by the patient corresponding to each item need to be added and divided by the number of items contained in that scale. A score lower or equal to 2 implies no distress, moderate distress is described by a score higher than 2 but lower or equal to 2.9, and severe distress is considered if the score is equal or greater than 3.

As mentioned before, the following parameters were also evaluated: socioeconomic and demographic data, including gender, ethnicity, place of residence, living conditions, employment status, and education.

The place of residence was sorted by living in the urban or rural area.

Living conditions were classified into the following categories: alone, with a spouse, with family, or in concubinage.

Employment status was determined from participants' self-reported information and grouped into the subsequent sections, namely unemployed, employed, retired, or student.

To evaluate education levels, we categorized individuals based on their last attended institution into five groups: no education, 8 years of education, 12 years of education, college, and post-secondary school.

Paraclinical assessment

The laboratory parameters analyzed were fasting plasmatic glycemia (FPG), glycated hemoglobin (HbA1c) level, serum creatinine, glutamic-oxaloacetic transaminase (GOT), glutamate pyruvate transaminase (GPT), total cholesterol (TC), high-density lipoprotein cholesterol (HDL-c), low-density lipoprotein cholesterol (LDL-c), and triglycerides (TGL). The HbA1c level was determined using the high-performance liquid chromatography (HPLC) method. LDL-cholesterol level was either calculated using the following formula: total cholesterol minus HDL-cholesterol minus triglycerides/5 if the triglycerides level was below 400 mg/dl or measured directly in the laboratory for triglycerides levels above 400 mg/dl.

Statistical analysis

Statistical analysis of the population was performed with the IBM SPSS Statistics for Windows, Version 19 (Released 2010; IBM Corp., Armonk, New York, United States). The continuous variables normally distributed were reported as mean±standard deviation (SD), while non-normal variables were described as median±interquartile range (IQR) or median±95% confidence interval (CI); the categorical variables were presented as absolute counts as well as percentages. Statistical significance was set at a 95% CI. Analysis of variance (ANOVA) was used for comparisons among groups for quantitative variables, while for categorical variables, the χ^2^ test was used. Forward conditional binary logistic regression was performed to identify the independent risk factors for diabetes distress.

## Results

Socioeconomic and demographic factors

Table 1 summarizes the general characteristics of patients. The study conducted included 165 Roma patients and 145 Caucasians; in both groups, most of them were women (63% and 58.6%, respectively). The majority of the patients (71%, N=103 in the Caucasian group and 73.9%, N=122 in the Roma group, respectively) had T2DM.

**Table 1:** General characteristics of the patients according to ethnicity The data has been represented as absolute counts ("N") and percentages ("%"). T1DM: type 1 diabetes mellitus; T2DM: type 2 diabetes mellitus

Variables		Caucasian patients (N=145)	Roma patients (N=165)
Gender	Men	41.4% (N=60)	37% (N=61)
	Women	58.6% (N=85)	63% (N=104)
Type of diabetes	T1DM	29% (N=42)	26.1% (N=43)
	T2DM	71% (N=103)	73.9% (N=122)
Place of residence	Urban area	75.2% (N=109)	43% (N=71)
	Rural area	24.8% (N=36)	57% (N=94)
Living conditions	Alone	15.9% (N=23)	9.7% (N=16)
	With spouse	51.7% (N=75)	33.9% (N=56)
	With family	31% (N=45)	55.2% (N=91)
	Concubinage	1.4% (N=2)	1.2% (N=2)
Employment status	Unemployed	6.2% (N=9)	20.6% (N=34)
	Employed	33.1% (N=48)	27.3% (N=45)
	Retired	57.9% (N=84)	48.5% (N=80)
	Student	2.8% (N=4)	3.6% (N=6)
Level of education	No education	0% (N=0)	4.8% (N=8)
	8 classes	16.6% (N=24)	57.6% (N=95)
	12 classes	37.9% (N=55)	19.4% (N=32)
	College	32.4% (N=47)	12.2% (N=20)
	Post-secondary school	13.1% (N=19)	6.1% (N=10)

Regarding the place of residence, most of the Roma patients lived in rural areas (57%, N=94), while 75.2% (N=109) of the Caucasian patients in urban areas.

With reference to their living conditions, a greater number of Roma patients (55.2%, N=91) lived with their family (parents and children), while more than half of the Caucasian patients (51.7%, N=75) lived with their spouses. There was a slight difference regarding the number of patients that lived alone, with a higher preponderance among the Caucasian patients compared to the Roma, and no discrepancy between the two groups concerning the number of patients living in concubinage.

As regards their employment status, among both groups, the majority of the patients were retired (57.9%, N=84 Caucasians versus 48.5%, N=80 Roma); there was a minor difference in employed participants between the two groups. A greater proportion of the Roma patients were unemployed compared to the Caucasian group (20.6%, N=34 versus 6.2%, N=9).

More than a third of the Caucasians graduated from high school (N=55, 37.9%), while nearly two-thirds (N=95, 57.6%) of the Roma patients graduated from general school. There were no patients in the Caucasian group that had no education, in comparison with the Roma population, where there were eight patients (4.8%).

Comparing the two groups, the Caucasian population had a slightly higher mean age (56.72±15.60 years) and median duration of diabetes (11.00±8.85 years) than the Roma group (Table 2).

Laboratory findings

Regarding glycemic control, the average HbA1c level was below 8% (7.66%±2.18) in the Caucasian group, and the mean fasting plasmatic glycemia was 161.42±73.57 mg/dl. Among the Roma patients were higher mean values of the HbA1c level, fasting plasmatic glucose level, total cholesterol, and LDL-c levels, which were statistically significant (Table 2).

**Table 2:** Mean values of the analyzed parameters according to ethnicity The data has been represented as mean±standard deviation (SD) and median±IQR (marked with "*"). The statistical significance was considered at a p-value < 0.05. HbA1c (%): glycated hemoglobin; FPG (mg/dl): fasting plasmatic glycemia; GOT (UI/l): glutamic oxaloacetic transaminase; GPT (UI/l): glutamic pyruvic transaminase; TC (mg/dl): total cholesterol; HDL-c (mg/dl): high-density lipoprotein cholesterol; LDL-c (mg/dl): low-density lipoprotein cholesterol; TGL (mg/dl): triglycerides; IQR: interquartile range

Parameter	Caucasian patients (N=145)		Roma patients (N=165)		p-value
Age (years)	56.72	15.6	54.50	14.54	0.198
Diabetes duration (years)	11.00*	8.85	8.50*	8.59	0.224
HbA1c (%)	7.66	2.18	8.22	2.53	0.133
FPG (mg/dl)	161.42	73.57	193.64	77.02	0.0001
Creatinine (mg/dl)	0.92	0.35	0.96	0.48	0.313
GOT (UI/l)	23.00*	25.38	22.00*	13.18	0.061
GPT (UI/l)	28.00*	26.47	24.50*	14.17	0.052
TC (mg/dl)	183.99	45.17	203.21	63.36	0.005
HDL-c (mg/dl)	53.73	16.57	51.84	16.37	0.344
LDL-c (mg/dl)	92.00*	41.87	101.00*	47.39	0.001
TGL (mg/dl)	148.00*	68.36	175.00*	84.47	0.089

Diabetes distress

Analyzing the average scores of diabetes distress according to the ethnicity of the patients, in the Caucasian group, women had a higher median total DDS (2.23, 95% CI 2.19-2.69) compared to men; the same trend is observed in all subscales of the diabetes distress score. A statistical significance was observed regarding interpersonal distress, with Caucasian women having a higher score than men (Table 3). With reference to the Roma patients, total DDS and all subscales´ scores were statistically significant, with Roma women having higher scores than men. Apart from this, a statistical significance was observed between ethnicity and diabetes distress scores, with the Roma population having higher median values than Caucasian patients (Table 4).

**Table 3:** Average scores of diabetes distress in Caucasian patients The data has been represented as a median±95% CI. The statistical significance was considered at a p-value < 0.05. *between Caucasian women and Caucasian men CI: confidence interval; DDS: diabetes distress scale

Diabetes distress scales	Caucasian women (N=85)		Caucasian men (N=60)		Caucasian patients (N=145)		p-value*
Total DDS	2.23	2.19-2.69	2.00	1.94-2.53	2.11	2.17-2.55	0.284
Emotional burden	2.20	2.11-2.64	1.80	1.82-2.41	2.00	2.07-2.47	0.187
Physician-related distress	2.25	2.22-2.72	2.00	1.92-2.52	2.25	2.17-2.57	0.196
Regimen-related distress	2.20	2.31-2.87	2.00	2.1-2.79	2.20	2.32-2.75	0.505
Interpersonal distress	2.00	1.97-2.57	1.66	1.79-2.44	2.00	1.99-2.42	0.0001

**Table 4:** Average scores of diabetes distress in Roma patients The data has been represented as median±95% confidence interval (CI). The comparison of the median value was performed using ANOVA. The statistical significance was considered at a p-value < 0.05. *between Roma women and Roma men; **between Caucasian and Roma patients ANOVA: analysis of variance; DDS: diabetes distress scale

Diabetes distress scales	Roma women (N=104)		Roma men (N=61)		Roma patients (N=165)		p-value*	p-value**
Total DDS	3.20	2.97-3.49	2.29	2.18-2.80	2.88	2.76-3.17	<0.001	<0.001
Emotional burden	3.00	2.77-3.29	2.20	2.03-2.64	2.60	2.57-2.98	0.001	0.001
Physician-related distress	3.25	2.92-3.47	2.25	2.11-2.78	2.75	2.70-3.14	0.001	<0.001
Regimen-related distress	3.60	3.28-3.83	2.80	2.48-3.18	3.20	3.07-3.51	0.001	<0.001
Interpersonal distress	2.66	2.77-3.42	1.66	1.88-2.62	2.33	2.53-3.04	0.001	0.001

In the study population, a great proportion of patients showed diabetes distress, with 24.8% (N=82) having moderate distress, and 29.7% (N=121) having severe distress. Regarding the total DDS, there was a predominance in the Caucasian group of patients without distress (61.7%, N=66 versus 38.3%, N=41 in the Roma population), while on the contrary, more Roma patients experienced severe distress compared to the opposite group (64.5%, N=78 versus 35.5%, N=43), the same trend being observed regarding the emotional burden, physician-related distress, regimen-related distress, and interpersonal distress. Overall, approximately a third of the patients included in the study reported severe distress observed in the emotional burden, physician-related, and interpersonal distress subscales of the DDS questionnaire, with 38.1% (N=118), 38.7% (N=120) and 29.7% (N=92), respectively, while nearly half of them (48.4%, N=150) described severe regimen-related distress (Table 5).

**Table 5:** Diabetes distress scores according to ethnicity The data has been represented as absolute counts ("N") and percentages ("%"). The statistical significance was considered at a p-value < 0.05.

Diabetes distress scales	Level of distress	Total (N=310)	Caucasian patients (N=145)	Roma patients (N=165)	p-value
Total distress score	Without distress	45.5% (N=107)	61.7% (N=66)	38.3% (N=41)	0.0001
	Moderate distress	24.8% (N=82)	43.9% (N=36)	56.1% (N=46)	
	Severe distress	29.7% (N=121)	35.5% (N=43)	64.5% (N=78)	
Emotional burden	Without distress	39% (N=121)	59.5% (N=72)	40.5% (N=49)	0.001
	Moderate distress	22.9% (N=71)	40.8% (N=29)	59.2% (N=42)	
	Severe distress	38.1% (N=118)	37.3% (N=44)	62.7% (N=74)	
Physician-related distress	Without distress	34.5% (N=107)	57% (N=61)	43% (N=46)	0.003
	Moderate distress	28.8% (N=26.8%)	50.6% (N=42)	49.4% (N=41)	
	Severe distress	38.7% (N=120)	35% (N=42)	65% (N=78)	
Regimen-related distress	Without distress	30.3% (N=94)	62.8%(N=59)	37.2% (N=35)	0.0001
	Moderate distress	21.3% (N=66)	53% (N=35)	47% (N=31)	
	Severe distress	48.4% (N=150)	34% (N=51)	66% (N=99)	
Interpersonal distress	Without distress	41.6% (N=120)	54.3% (N=70)	45.7% (N=59)	0.026

In the study population with severe diabetes distress scores, the average age of the patients was higher in patients with severe emotional burden scores; the median value of diabetes duration was higher in patients with severe emotional burden and physician-related distress scores. Regarding glycemic control, the mean value of HbA1c level was higher in patients with severe regimen-related distress score (8.55±2.61%). Moreover, the mean FPG level (194.30±79.79 mg/dl) was higher in patients with severe physician-related distress scores (Table 6).

**Table 6:** Mean values of the analyzed parameters according to severe DDS The data has been represented as mean±standard deviation (SD) and median±interquartile range (IQR) (marked with "**"). The statistical significance was considered at a p-value < 0.05. HbA1c (%): glycated hemoglobin; FPG (mg/dl): fasting plasmatic glycemia; GOT (UI/l): glutamic oxaloacetic transaminase; GPT (UI/l): glutamic pyruvic transaminase; TC (mg/dl): total cholesterol; HDL-c (mg/dl): high-density lipoprotein cholesterol; LDL-c (mg/dl): low-density lipoprotein cholesterol; TGL (mg/dl): triglycerides

Parameters	Severe diabetes distress scores							
	Emotional burden		Physician-related distress		Regimen-related distress		Interpersonal distress	
Age (years)	55.63	14.34	54.40	15.45	54.45	14.84	52.53	14.09
Diabetes duration (years)	14.00**	9.13	14.00**	9.02	12.00**	8.85	12.00**	7.93
HbA1c (%)	8.43	2.44	8.43	2.76	8.55**	2.61	8.21	1.95
FPG (mg/dl)	191.85	79.61	193.85**	77.69	194.30**	79.79	185.92	80.15
Creatinine (mg/dl)	0.99	0.52	1.02	0.54	1.01	0.55	1.06	0.63
GOT (UI/l)	21.00**	14.32	22.00**	14.67	22.00**	25.60	22.00**	18.10
GPT (UI/l)	24.00**	14.58	26.00**	14.51	26.61**	25.94	27.00**	15.92
TC (mg/dl)	199.26	59.38	198.86	58.41	197.95	51.30	200.16	53.16
HDL-c (mg/dl)	54.32	18.76	54.37	18.89	53.49	18.92	54.29	22.18
LDL-c (mg/dl)	97.00**	47.71	93.80**	48.24	98.00**	49.90	100.20**	53.64
TGL (mg/dl)	172.00**	86.48	178.00**	83.20	173.00**	82.71	172.00**	88.83

In patients with severe total DDS, there is a statistical significance between ethnicity and the mean value of FPG, with the Roma patients having higher levels of fasting blood glucose compared to Caucasians (Table 7).

**Table 7:** Severe total DDS The data has been represented as mean±standard deviation (SD) and median±interquartile range (IQR) (marked with "*"). The statistical significance was considered at a p-value < 0.05. HbA1c (%): glycated hemoglobin; FPG (mg/dl): fasting plasmatic glycemia; GOT (UI/l): glutamic oxaloacetic transaminase; GPT (UI/l): glutamic pyruvic transaminase; TC (mg/dl): total cholesterol; HDL-c (mg/dl): high-density lipoprotein cholesterol; LDL-c (mg/dl): low-density lipoprotein cholesterol; TGL (mg/dl): triglycerides; DDS: diabetes distress scale

Parameters	Severe total DDS				
	Caucasian patients (N=145)		Roma patients (N=165)		p-value
Age (years)	56.16	15.03	54.56	15.15	0.362
Diabetes duration (years)	18.50*	8.06	8.00*	8.31	0.063
HbA1c (%)	7.72	2.20	8.28	2.59	0.148
FPG (mg/dl)	169.54	74.64	192.98	78.80	0.009
Creatinine (mg/dl)	0.98	0.46	1.05	0.65	0.174
GOT (UI/l)	23.00*	17.61	21.00*	12.58	0.474
GPT (UI/l)	27.00*	17.75	23.00*	12.33	0.620
TC (mg/dl)	189.96	53.70	201.17	60.12	0.108
HDL-c (mg/dl)	51.20	12.75	54.99	19.74	0.062
LDL-c (mg/dl)	91.50*	48.53	101.80*	49.62	0.606
TGL (mg/dl)	174.50*	73.25	178.00*	89.42	0.351

Factors contributing to diabetes distress

We conducted univariate analysis on gender, age, ethnicity, place of residence, living conditions (living alone), employment status (unemployed), level of education (without education and college graduates), duration of diabetes (over 10 years of evolution), type of diabetes, HbA1c value (above 8%). The variables significantly associated with severe diabetes distress score were included in a binary logistic regression analysis (forward conditional).

Multivariate analysis of factors contributing to diabetes distress

Factors associated with severe diabetes distress scores are reported in Table 8. It was demonstrated that the lack of education, a higher diabetes evolution, and a higher HbA1c level (above 8%) have influenced the risk of severe DDS in the Caucasian group, while in the Roma patients, employment status (unemployed) represents a risk factor for severe DDS.

**Table 8:** Factors associated with severe DDS Logistic regression coefficient and odds ratio (95% CI). The statistical significance was considered at a p-value < 0.05. SE: standard error; OR: odds ratio; CI: confidence interval; DDS: diabetes distress scale; B: standard beta coefficient

Variables	B	SE	p-value	OR	95% CI	
					Lower	Upper
In Caucasian patients						
HbA1c level (above 8%)	0.302	0.139	0.030	1.35	1.029	1.77
Level of education (no education)	1.067	0.493	0.030	2.90	1.106	7.645
Diabetes duration (above 10 years)	-1.123	0.465	0.016	0.325	0.131	0.809
In Roma patients						
Employment status (unemployed)	-0.954	0.446	0.032	0.385	0.161	0.924

## Discussion

We used data from a Romanian sample of adults with T2DM predominantly and analyzed the diabetes distress and possible factors influencing it comparing the Roma patients with a control group of Caucasian patients.

Regarding the socioeconomic and demographic characteristics, in contrast with the control group, the Roma population lived in the rural area, with their families and were unemployed. Data from the medical literature suggests corresponding characteristics, lower employment rates, and higher overcrowded housing conditions being observed in the Roma population from Hungary, Greece, or Serbia [11-14]. In the present study, a great proportion of the Roma population graduated from eight classes. These findings are consistent with the results of other papers which revealed that almost 85% only had primary school education [10,15].

In Romania, a study that included participants without diabetes, there was no significant difference between Roma and non-Roma referring to the marital status, but there were significant disparities regarding the level of education (secondary school completion for Roma versus high school graduation for the general population), household capacity (5.3 individuals for Roma versus 4.2 individuals for non-Roma), and literacy rate (61% literate Roma versus 97.4% literate non-Roma). Assessing the employment rates between the groups, there was a slight difference, with a percentage of 26.6% in Roma versus 32.4% in non-Roma [16]. Measuring the HRQoL of the Roma population in Romania, in participants without diabetes, the Roma community had a lower level of HRQoL than the general population; the analysis was made using the EQ-5D-5L questionnaire [17]. Apart from this, Pappa et al. analyzed the socioeconomic factors and HRQoL among the Roma population in Greece and observed that 90.6% of them declared stable housing conditions, 71.2% reported more than five roommates, and a significant rate of 12.5% reported a monthly income of more than 1000 euros. However, approximately 39% had no access to drinkable water, electricity, or an indoor bath. Moreover, higher scores of HRQoL assessed using the Short Form (SF)-36 Health Survey were observed in younger participants, and in men [18]. The SF-36 Health Survey is a 36-item self-administered instrument broadly used to measure HRQoL. The questionnaire comprises eight scales, including physical functioning (PF), role limitations due to physical problems (RP), bodily pain (BP), general health (GH), vitality (VT), social functioning (SF), role limitations due to emotional problems (RE), and Mental Health (MH). Each scale is rated from 0 to 100, where higher scores indicate a better perception of health. Previous studies in Greece have validated the SF-36 questionnaire [18].

According to the Predatorr study, the prevalence of diabetes in Romania was 11.6%, with a higher percentage observed in men than in women [19]. In a study from Romania that compared the general Romanian population to the Roma population, the majority of the study population consisted of Roma patients; moreover, a greater part of the participants were women; the prevalence of diabetes in Caucasians was 14.6%, while among the Roma patients was 11.7% [10]. In our study, our findings are consistent with the previously mentioned paper, with a higher percentage of women being included in the study, probably due to a higher number of women being hospitalized during that period; apart from this, although the Roma minority represents only a small percentage of the Romanian population, a larger group of Roma patients being recruited in contrast with Romanian Caucasians could be explained by the fact that our hospital is more accessible to them, being a tertiary hospital providing diabetes care and located to the peripheral area of the city.

Regarding diabetes distress, among the Roma population, the findings of our study are significant as the medical literature is deficient in this subject. Our paper revealed a higher prevalence of diabetes distress among the Roma minority compared to the Caucasian group, the majority of them associating severe diabetes distress. There are no data in Romania concerning the distress among the Roma population with diabetes.

Compared to previous studies that included mostly ethnic minorities, ethnicity was significantly associated with a higher total distress score [20]. In Caucasian patients, the odds of having severe diabetes distress score were higher in patients who had no school, as shown in a paper by Ratnesh et al. [21].

As concluded by Kokoszka et al., in a study from Poland that included patients with T2DM, women had higher diabetes distress analyzed with PAID [22], consistent with the findings in our study.

Moreover, a study from Italy revealed that in patients with T2DM, high diabetes-related distress score assessed with PAID was associated with living conditions (living alone) and low education level [23]; similarly, in our study, in the Caucasian group, lack of education has also influenced the risk of severe DDS.

Data from a study conducted in Chinese adults with T2DM, the prevalence of diabetes distress was 34.64%. Approximately one-third (24.29%) of the subjects had moderate distress, while only 10.35% had severe distress. The results from our study are similar regarding the proportion of moderate distress (24.8%), but a higher proportion of patients with severe distress was observed (29.7%). Among Chinese young adults with T2DM, 33.67% had moderate diabetes distress and a significantly higher proportion had severe distress (57.14%); apart from this, men had higher diabetes distress scores compared to women [24]. On the contrary, in our study, both Caucasian and Roma women had higher total DDS than men.

In a paper that analyzed diabetes distress using the PAID-20 questionnaire among adult patients with T1DM, 21.7% reported high levels of distress; apart from this, there were identified associations between distress and gender, age, non-European origin of the patients, primary school level, unemployment, lower evolution of diabetes and higher HbA1c levels [25]. Similarly, in our study, 29.7% of the patients showed a severe total diabetes distress score, which was statistically associated with higher HbA1c levels. Schmidt et al. emphasized the same findings when assessing the differences between a native Dutch population and immigrant ethnic minorities such as Moroccan, Turkish, or Surinamese. Higher levels of diabetes distress measured with the PAID-5 questionnaire were more frequent among the ethnic minorities compared to the native Dutch responders; furthermore, it was reported that the odds of diabetes distress were higher for Moroccans compared to the native Dutch [26].

Limitations of the study

Regarding the limitations of our study, we acknowledge the rather small sample size of patients could raise concerns about generality. Moreover, the enrollment of the participants was conducted in only one tertiary care hospital, during the COVID-19 pandemic period and although our results are consistent with those of larger studies that compared the general population with ethnic minorities, there are limited data on Roma patients, therefore we recognize the need for further research. Another limitation of our study could result from the fact that participants may have provided inaccurate or biased responses, consciously or unconsciously, particularly less educated patients, due to social desirability or other factors.

## Conclusions

Taking the above into consideration, our study revealed that 24.8% of the patients selected from a tertiary hospital providing diabetes care had moderate distress, and 29.7% had severe distress. A higher percentage of Roma patients showed severe distress in contrast with the corresponding group of Caucasians, where the majority did not present distress. These results are substantial as they emphasize the need to include the evaluation of diabetes distress in clinical practice to facilitate the early initiation of intervention measures and further investigate the implications of ethnicity on the level of distress.

## References

[1] (2023). Initial management of hyperglycemia in adults with type 2 diabetes mellitus - UpToDate. https://www.uptodate.com/contents/initial-management-of-hyperglycemia-in-adults-with-type-2-diabetes-mellitus.

[2] (2023). Overview of general medical care in nonpregnant adults with diabetes mellitus - UpToDate. https://www.uptodate.com/contents/overview-of-general-medical-care-in-nonpregnant-adults-with-diabetes-mellitus.

[3] Hessler DM, Fisher L, Polonsky WH (2017). Diabetes distress is linked with worsening diabetes management over time in adults with type 1 diabetes. Diabet Med.

[4] Hislop AL, Fegan PG, Schlaeppi MJ, Duck M, Yeap BB (2008). Prevalence and associations of psychological distress in young adults with type 1 diabetes. Diabet Med.

[5] American Diabetes Association Professional Practice Committee (2024). Summary of revisions: Standards of care in diabetes-2024. Diabetes Care.

[6] Polonsky WH, Fisher L, Earles J, Dudl RJ, Lees J, Mullan J, Jackson RA (2005). Assessing psychosocial distress in diabetes: development of the diabetes distress scale. Diabetes Care.

[7] Akter J, Islam RM, Chowdhury HA (2022). Psychometric validation of diabetes distress scale in Bangladeshi population. Sci Rep.

[8] Mocan A, Băban A (2015). An useful tool for diabetes emotional distress assessment: validation of the Romanian version of diabetes distress scale. RomJ Diabetes Nutr Metab Dis.

[9] (2022). Rezultate 2021 - Institutul Național de Statistică. https://www.recensamantromania.ro/rezultate-rpl-2021/rezultate-definitive-caracteristici-etno-culturale-demografice/.

[10] Enache G, Rusu E, Ilinca A (2018). Prevalence of overweight and obesity in a Roma population from Southern Romania - Calarasi county. Acta Endocrinol (Buchar).

[11] Llanaj E, Vincze F, Kósa Z, Sándor J, Diószegi J, Ádány R (2020). Dietary profile and nutritional status of the Roma population living in segregated colonies in Northeast Hungary. Nutrients.

[12] Petraki I, Kalpourtzi N, Terzidis A (2021). Living in Roma settlements in Greece: self-perceived health status, chronic diseases and associated social determinants of health. Int J Environ Res Public Health.

[13] Janevic T, Jankovic J, Bradley E (2012). Socioeconomic position, gender, and inequalities in self-rated health between Roma and non-Roma in Serbia. Int J Public Health.

[14] Kasabji F, Alrajo A, Vincze F, Kőrösi L, Ádány R, Sándor J (2020). Self-declared Roma ethnicity and health insurance expenditures: a nationwide cross-sectional investigation at the general medical practice level in Hungary. Int J Environ Res Public Health.

[15] Weiss E, Japie C, Balahura AM, Bartos D, Badila E (2018). Cardiovascular risk factors in a Roma sample population from Romania. Rom J Intern Med.

[16] Powell Doherty R, Telionis PA, Müller-Demary D (2017). A survey of quality of life indicators in the Romanian Roma population following the 'Decade of Roma Inclusion'. F1000Res.

[17] Robinson T, Oluboyede Y, Vale L, Olariu E (2022). Differences in health-related quality of life between the Roma community and the general population in Romania. J Patient Rep Outcomes.

[18] Pappa E, Chatzikonstantinidou S, Chalkiopoulos G, Papadopoulos A, Niakas D (2015). Health-related quality of life of the Roma in Greece: the role of socio-economic characteristics and housing conditions. Int J Environ Res Public Health.

[19] Mota M, Popa SG, Mota E (2016). Prevalence of diabetes mellitus and prediabetes in the adult Romanian population: PREDATORR study. J Diabetes.

[20] German J, Kobe EA, Lewinski AA (2023). Factors associated with diabetes distress among patients with poorly controlled type 2 diabetes. J Endocr Soc.

[21] Ratnesh Ratnesh, Shivaprasad KS, Kannan S, Khadilkar KS, Sravani GV, Raju R (2020). Identifying the burden and predictors of diabetes distress among adult type 2 diabetes mellitus patients. Indian J Community Med.

[22] Kokoszka A, Pacura A, Kostecka B, Lloyd CE, Sartorius N (2022). Body self-esteem is related to subjective well-being, severity of depressive symptoms, BMI, glycated hemoglobin levels, and diabetes-related distress in type 2 diabetes. PLoS One.

[23] Pintaudi B, Lucisano G, Gentile S (2015). Correlates of diabetes-related distress in type 2 diabetes: findings from the benchmarking network for clinical and humanistic outcomes in diabetes (BENCH-D) study. J Psychosom Res.

[24] Hu Y, Li L, Zhang J (2020). Diabetes distress in young adults with type 2 diabetes: a cross-sectional survey in China. J Diabetes Res.

[25] Hernar I, Cooper JG, Nilsen RM (2024). Diabetes distress and associations with demographic and clinical variables: a nationwide population-based registry study of 10,186 adults with type 1 diabetes in Norway. Diabetes Care.

[26] Schmidt CB, Potter van Loon BJ, Torensma B, Snoek FJ, Honig A (2017). Ethnic minorities with diabetes differ in depressive and anxiety symptoms and diabetes-distress. J Diabetes Res.

